# The Effects of Tight Clothing

**Published:** 1890-01

**Authors:** 


### THE EFFECTS OF TIGHT CLOTHING.

Now that rational ideas as to dress have acquired a definite place in public esteem, it may be imagined that the practice of tight lacing, and customs of a like nature, if known at all, are not what they used to be. A case of sudden death lately reported from Birmingham proves that it is still too early to indulge in such illusory ideas. The deceased, a servant girl of excitable temperment, died suddenly in an epileptoid fit and the evidence given before the coroner respecting her death attributed the fatal issue to asphyxia, due in a great measure to the fact that both neck and waist were unnaturally constricted by her clothing, the former by a tight collar, the latter by a belt worn under the stays. We have here certainly those very conditions which would lead us to expect the worst possible consequences from a convulsive seizure. There is no organ of the body whose free movement is at such times more important than the heart. Yet here we find, on the one hand, its movement hampered by a tight girdle so placed that it could with difficulty be undone at a critical moment ; on the other a contrivance admirably adapted to allow the passage of the blood to the brain, while impeding its return. This is no isolated case as regards its essential character, though, happily, somewhat singular in its termination. Minor degrees of asphyxiation, we fear, are still submitted to by many of the self-torturing children of vanity. The tight corset and the high heel still work mischief on the bodies of their devoted wearers. Taste and reason, indeed combine to deprecate their injurious and vulgar bondage, and by no means unsuccessfully. Still the evil maintains itself. Cases like that above mentioned ought to, if they do not, open the eyes of some of the self-worshippers of the other sex who heedlessly strive by such means to excel in a sickly grace. We would strongly impress on all of this class the fact that beauty is impossible without health, and would advise them in the name of taste as well as comfort, to avoid those methods of contortion, one and all, by which elegance is only caricatured, and health may be painfully and permanently injured.—*London Lancet.*

